# Intergenerational Transmission of Maternal Childhood Maltreatment Prior to Birth: Effects on Human Fetal Amygdala Functional Connectivity

**DOI:** 10.1016/j.jaac.2023.03.020

**Published:** 2023-05-26

**Authors:** Marion I. van den Heuvel, Catherine Monk, Cassandra L. Hendrix, Jasmine Hect, Seonjoo Lee, Tianshu Feng, Moriah E. Thomason

**Affiliations:** Tilburg University, Tilburg, the Netherlands; New York State Psychiatric Institute, New York. Columbia University, New York, NY; NYU Langone Health, New York; University of Pittsburgh, Pennsylvania, Pittsburgh; New York State Psychiatric Institute, New York. Columbia University, New York, NY; New York State Psychiatric Institute, New York. Research Foundation for Mental Hygiene, Inc., New York; NYU Langone Health, New York. Neuroscience Institute, NYU Langone Health, New York

**Keywords:** brain, fetal, childhood maltreatment, maternal, intergenerational transmission

## Abstract

**Objective:**

Childhood maltreatment (CM) is a potent risk factor for developing psychopathology later in life. Accumulating research suggests that the influence is not limited to the exposed individual but may also be transmitted across generations. In this study, we examine the effect of CM in pregnant women on fetal amygdala–cortical function, prior to postnatal influences.

**Method:**

Healthy pregnant women (N = 89) completed fetal resting-state functional magnetic resonance imaging (rsfMRI) scans between the late second trimester and birth. Women were primarily from low socioeconomic status households with relatively high CM. Mothers completed questionnaires prospectively evaluating prenatal psychosocial health and retrospectively evaluating trauma from their own childhood. Voxelwise functional connectivity was calculated from bilateral amygdala masks.

**Results:**

Connectivity of the amygdala network was relatively higher to left frontal areas (prefrontal cortex and premotor) and relatively lower to right premotor area and brainstem areas in fetuses of mothers exposed to higher CM. These associations persisted after controlling for maternal socioeconomic status, maternal prenatal distress, measures of fetal motion, and gestational age at the time of scan and at birth.

**Conclusion:**

Pregnant women’s experiences of CM are associated with offspring brain development in utero. The strongest effects were found in the left hemisphere, potentially indicating lateralization of the effects of maternal CM on the fetal brain. This study suggests that the time frame of the Developmental Origins of Health and Disease research should be extended to exposures from mothers’ childhood, and indicates that the intergenerational transmission of trauma may occur prior to birth.

Increasingly, neuropsychiatric disorders are considered neurodevelopmental in etiology,^1^ with a range of environmental exposures, such as lead exposure in early life and non-contingent rearing, significantly contributing to altered brain–behavior trajectories and risk for psychopathology.^2-5^ Parental experiences from parents’ own childhood have also been established as a risk factor for psychopathology in the next generation.^6^ Notably, effects of childhood maltreatment (CM) on the subsequent generation can be long ranging, with persistent depressive symptoms shown to extend into the fourth decade of life in the offspring of mothers with prior CM.^7^ The public health relevance of intergenerational transmission of CM is thus considerable, as there is potential for early interventions, preferably before conception, to ameliorate the negative psychiatric sequelae of CM for multiple generations.

The underlying mechanisms of intergenerational transmission are still largely unclear. One explanation is that exposure to CM may have a negative impact on subsequent parenting practices, which may place children at greater risk.^8^ Another possibility is that CM may have an enduring influence on maternal psychobiology, which may influence the perinatal and early-rearing environment into which a child is born.^9,10^ Developmental Origins of Health and Disease (DOHaD) research has demonstrated an even earlier time frame for significant environmental influence, namely, during the prenatal period. The DOHaD model suggests that intergenerational transmission of maternal CM effects may begin prior to birth through long-term alterations in materal—or paternal—biology affecting gestational biology and fetal brain development.^9,10^ In the DOHaD model, the fetus “adapts” to his or her environment with “programmed” changes in their biology aimed at ensuring survival. Support for such intergenerational transmission comes from animal studies showing increased oxidative stress indicators in oocyte milieu in obese or stressed females and epigenetic alterations in the sperm of male rodents exposed to early life stress.^11,12^

Although human research on the DOHaD model for maternal CM on brain development of the fetus is still lacking, recent studies in newborns show interesting initial results. In novel imaging work, Moog *et al*.^13^ reported that 4-week old infants of mothers exposed to CM had a 6% reduction intracranial volume. In addition, Hendrix *et al*.^14^ found that newborns of mothers who reported higher emotional neglect during their own childhood had stronger fuctional connectivity between the amygdala and the ventomedial prefrontal cortex (vmPFC) and dorsal anterior cinculate cortex (dACC). This association was specific to emotional neglect and was not explained by maternal distress during pregnancy. More recently, Khoury *et al*.^15^ reported an association between maternal CM and lower brain volumes of overall gray matter and the amygdala over the first 2 years postpartum. The smaller amygdala volume was found only at older ages. Taken together, these studies provide initial evidence for altered brain development in infants of mothers who experienced CM in their childhood. By studying the infant brain proximal to birth, these studies minimized—but did not eliminate—potential influences of the postnatal environment. A more focused investigation of maternal CM effects on prenatal brain development is needed to provide evidence of the potential of prenatal programming in relation to maternal childhood maltreatment history.

A key brain region in the study of maltreatment exposure and altered neurodevelopment is the amygdala and its related brain network. Several studies of trauma-exposed youth report variation in amygdala based fear circuitry, mostly in the amygdala–prefrontal connection.^16,17^ Altered amygdala connectivity, in turn, was found to be associated with behavioral issues and mood disturbances.^18^ Furthermore, studies focusing on the effects of maternal CM on infant brain development either reported on amygdala functional connectivity^14^ or reported alterations in the volume of the amygdala.^13,15^ In line with the DOHaD model, these alterations of amygdala functional connectivity could be see as developmental adaptations in response to early adversity. In a seminal study by Gee *et al*.,^19^ for instance, children with a history of early adversity showed more mature amygdala–mPFC connectivity, which was associated with reduced anxiety symptoms (even though anxiety scores were still higher in the exposed children as compared to the non-exposed children). Together, these studies indicate that the amygdala and its connections are implicated in early adversity, in both the current and next generations. In this report, we focus on amygdala–cortical functional connectivity as a target for the effects of inter-generational transmission of trauma prior to birth.

The current study aimed to examine the effects of maternal CM on fetal amygdala–cortical functional connectivity in late pregnancy in a predominately low-resource, Black American sample. Given that the majority of studies examining CM recruit participants from predominately White, highly educated, low-risk communities, resulting in deficient representation of individuals from low-resource and racial and ethnic minority groups, we recruited from a low-resource, high-risk population. In addition, rates of maltreatment are usually high in these populations, providing a sample with a wider variety of risk.^20^ Because there are sex differences in fetal and child brain development,^21,22^ prenatal programming,^23^ and rates of neuropsychiatric disorders,^24^ we examined sex as a moderator of the influence of maternal CM on fetal functional connectivity. All models controlled for maternal distress during pregnancy to better isolate the influence of maternal CM.

## METHOD

### Participants

A total of 221 pregnant women were recruited between the 18^th^ and 36^th^ gestational weeks into a longitiudinal cohort study based in Detroit, Michigan. Only pregnant women >18 years of age, native English speaking, with singleton pregnancy, were eligible to participate. Only fetuses with normal fetal brain anatomy, as assessed by ultrasound and magnetic resonance imaging (MRI) examination, were included in the study. Normal fetal brain anatomy was defined as having no detected anomalies in the fetal brain by the referring physician or the research team. Fetal gestational age (GA) was checked by a referring physician using ultrasound examination at the MRI visit. At age 3 years, scanned mothers were invited for a child play visit at the laboratory, at which point retrospective maternal childhood maltreatment history information was obtained as part of a larger questionnaire battery.

For the present analyses, we excluded cases with missing maltreatment history data (n = 51). Fetal scans of low image quality due to large artifacts (eg, air–tissue susceptibility) or excessive movement (n = 46) and scans with different scanning parameters (n = 6) were also excluded. Other exclusionary criteria were the presence of prenatal or birth complications (ie, premature rupture of membranes, intrauterine growth restriction, preeclampsia; n = 6), extreme preterm birth (<34 weeks of pregnancy; n = 11), and extremely low birthweight after controlling for gestational age (*z* scores of +2 or −2; n = 4).^25^ Finally, cases with too few artifact-free frames (<100) were excluded from the study (n = 5). These exclusions resulted in a final sample of 89 mother–fetus dyads (34 female fetuses), mean fetal age (at scan) 32.55 weeks (SD = 4.14; range 20.5739.57 weeks) for the analyses performed here. The resulting sample consisted of 42% of fetal cases in the original sample. This is a typical attrition rate, given the complexity of fetal scanning and a 3-year postnatal follow-up.^26^

All study procedures were approved by the Human Investigation Committee of Wayne State University, Detroit, MI, and all women provided written informed consent prior to participation. Sociodemographic characteristics were collected via online questionnaires (Qualtrics) during the pregnancy functional MRI (fMRI) visit. This included information on drug use during pregnancy (smoke exposure and alcohol consumption), which was measured as part of a health questionnaire designed for this study. Birth outcome data were collected from medical records. Sociodemographic characteristics and birth outcomes of the included mother-fetus dyads (N = 89) are provided in Table 1.

### Childhood Maltreatment and Psychosocial Health Measures

#### Maternal Maltreatment.

Maternal exposure to CM was obtained using the Childhood Trauma Questionnaire (CTQ),^27^ a 28-item self-report questionnaire that retrospectively assesses 5 types of childhood maltreatment: emotional, physical, and sexual abuse, and emotional and physical neglect. Each maltreatment type is assessed with 5 items measured on a 5-point Likert-type scale, with total scores for each subscale ranging from 5 to 25. The CTQ has excellent criterion-related validity (ie, internal consistency of α = 0.91).^27^ Mother completed the CTQ during the age 3 years laboratory visit, as part of a larger questionnaire battery.

Total CTQ score was computed by summing all subscale scores, with higher scores indicating greater CM severity. The total score was log-transformed to adjust for skewness. Analyses used continuous scores to mitigate data loss associated with data dichotomization. However, dichotomized CTQ scores were computed to characterize the prevalence of maltreatment in our sample. Following prior studies,^13^ dichotomized CTQ scores were based on suprathreshold exposure to at least 1 type of CM (CM+) compared to no or low exposure (CM−). CTQ thresholds for moderate to severe emotional, physical, and sexual abuse are ≥13, ≥10, and ≥8, respectively. Thresholds for emotional and physical neglect are ≥15 and ≥10.^27^ Missing values were mean imputed only when no more than 1 item was missing per subscale.

#### Maternal Prenatal and Postnatal Distress.

Maternal negative affect and distress during pregnancy were measured using 5 scales that assessed mood symptoms and indices of stress: the Center for Epidemiological Studies Depression Scale (CES-D),^28^ the State Trait Anxiety Inventory (STAI; only the state measures were collected, not trait),^29^ the Penn State Worry Questionnaire (PSWQ),^30^ the 10-item Perceived Stress Scale (PSS-10),^31^ and the Satisfaction with Life Scale (SWLS).^32^ Each measure addresses different categories of negative affectivity and stress in pregnant women. To reduce the number of tests and to extract the overlapping variance of these constructs, we combined these measures using exploratory factor analysis, following prior reports.^26,33^ The resulting single factor, hereafter referred to as the Negative Affectivity and Stress Factor (NASF), was used to quantify maternal prenatal distress and negative affect. The NASF was standardized, meaning that scores of 0 indicate average distress, whereas positive scores indicated stress higher than average and negative scores lower than average. A graphical representation of our factor score, including factor loadings, is presented in Figure S1, available online. Postnatally, at the visit 3 years after birth, mothers again completed measures of distress. Here, they reported on anxiety (STAI) and depression (CES-D).

#### Maternal Health Practices During Pregnancy.

Maternal smoke and alcohol exposure during pregnancy were measured using a self-report questionnaire about health behaviors.^34^ For smoking exposure, we examined both smoking (self) and exposure to second-hand smoke. For alcohol use, we computed a binary composite score based on 4 questions about drinking alcohol during pregnancy (eg, Have 5 or more alcoholic drinks per day (0 = no, 1 = yes); Limit my intake of alcohol (0 = no alcohol, 1 = alcohol); Drink alcohol until intoxicated (0 = no alcohol, 1 = alcohol); Drink alcohol excessively (0 = no alcohol, 1 = alcohol)). Mothers scored a “0” when not drinking any alcohol during pregnancy. For illustration purposes, we also report frequencies for problematic alcohol consumption during pregnancy (ie, Have 5 or more alcoholic drinks per day).

### MRI Data Acquisition

Fetal functional brain imaging was performed with a Siemens Verio 70-cm open-bore 3-Tesla scanner with a lightweight abdominal Siemens Flex Coil (Siemens, Munich, Germany). Resting-state fMRI data were acquired using a gradient echo planar imaging sequence: TR/TE 2000/30 milliseconds, flip angle 80°, 360 frames, axial 4-mm slice thickness, voxel size 3.4 × 3.4 × 4 mm^3^, repeated twice. Between 12 and 24 minutes of fetal resting-state fMRI data were collected per participant. The average system derived estimates for specific absorption rate was 0.22 watts per kilogram (SD = 0.06).

### Resting-State fMRI Data Preprocessing

Functional MRI preprocessing followed previously published methodology.^35^ Briefly, periods of fetal quiescence were manually identified using an FSL image viewer. Periods of low fetal motion were retained for analyses, resulting in exclusion of 37% of data and retention of an average of 173 frames, or 5.76 minutes (range = 103-344; SD = 53) per participant. Fetal brain masks corresponding to each epoch of low fetal motion were manually generated using Brainsuite.^36^ Masks were used for segmentation, which was followed by reorientation, realignment, and normalization to a 32-week fetal brain template^37^ using Statistical Parametric Mapping (SPM8) software implemented in MATLAB. Normalized images from each segment were then concatenated into one run, realigned, and smoothed with a 4-mm FWHM Gaussian kernel.

### Functional Connectivity Analyses

The fetal bilateral amygdala region of interest (ROI) was defined by first manually tracing the left hemisphere amygdala of a 32-week fetal brain template^37^ co-registered with the normalized fetal fMRI data. The left hemisphere trace was generated using the Multi-image Analysis GUI (MANGO; Research Imaging Institute, UT Health Science Center at San Antonio, TX; http://ric.uthscsa.edu/mango/) and added to a mirrored image in the contralateral hemisphere (see Figure S2, available online) to complete the ROI. The CONN functional connectivity toolbox (v14n)^38^ was used to generate voxel-level bi-lateral amygdala resting state functional connectivity (RSFC) maps for each subject. CONN processing included linear detrending, nuisance regression using aCompCor of 5 principal components extracted from a 32-week fetal atlas white matter and CSF mask, 6 head motion parameters, and band-pass filtering at 0.008 to 0.09 Hz.

Next, 2 second-level analyses were performed in SPM12: (1) 1-sample *t* tests to map fetal amygdala neurocircuitry, and (2) linear regression with maternal maltreatment severity as a predictor and gestational age at scan as a covariate. Resulting *t* maps were transformed into enhanced Z maps using probabilistic threshold free cluster enhancement (pTFCE).^39^ pTFCE integrates cluster information to provide voxel-level statistical inference in a probabilistic manner based on the Bayes rule, increasing sensitivity while also providing appropriate control for false-positive results, and is a recommended, developmentally sensitive strategy for improving reliability in the context of multiple comparisons.^40^ Images were subsequently thresholded at *p* < .05 (uncorrected), *k* > 20. A more liberal threshold was used in comparison with adult fMRI analyses, because the the fetal brain is much smaller, resulting in much fewer voxels. The “effective resolution” is therefore smaller as compared to adult neuroimaging (for a discussion, see van den Heuvel and Thomason^41^). RSFC values were extracted from 2-mm radius spheres at the peak voxel of each cluster that survived correction, using the Response Exploration package for Matlab (REX),^42^ for subsequent analyses. If clusters had several peaks (ie, in larger clusters), we extracted spheres for all peaks.

### Statistical Approach

RSFC values were extracted for ROIs that survived multiple comparisons correction. Individual fetal connectivity values were imported into SPSS (version 24.0.0.2, IBM Corp.) to test potential factors contributing to observed effects and to perform sensitivity analyses. First, we tested whether the association between maternal CM severity and fetal RSFC remained significant after controlling for additional confounders. Based on prior studies,^13,14,43^ we selected maternal NASF, maternal age at scan, maternal race, maternal education, family income, maternal smoke exposure during pregnancy, and fetal sex as confounders. Because very few mothers reported smoking (self) and alcohol use during pregnancy, these were not taken into account in the model. In addition, we controlled for translational and rotational motion values and frame count. It should be noted that fetal GA at scan was already taken into account in the SPM analyses described above. Because the fetal brain grows very rapidly, gestational age is a very important confounder in our analyses. We therefore partialled it out before making decisions regarding which brain areas to extract for further analyses. Second, the differential effect of CM by fetal sex on fetal RSFC was tested by adding the interaction terms between fetal sex and maternal CM to the model. All regressions were run with 5,000 bootstrapped samples and a *p* threshold of *p* = .05.

We also performed sensitivity analyses to address potential effects of outliers and fetal motion on our results. First, RSFC and CTQ values were identified by taking ±3 SD as a cutoff. Tested models were refitted with outliers excluded and rerun. There was 1 outlier for the CTQ, with a very high score of 113 (greater than +3 SD above the mean). Furthermore, there was 1 outlier identified for left visual RSFC. Second, we re-ran our analyses using the dichotomized CTQ scores to see whether the effects remained constant. Third, we computed Pearson correlations between brain quality measures (translational and rotational movement and the frame count) and our outcome variables to ensure that motion differences were not confounding observed effects. Finally, we also checked whether controlling for maternal postnatal stress at 3 years postpartum altered the results, as childhood maltreatment was reported by the mothers at the 3-year postpartum visit and may be influenced by maternal current distress levels. This was performed by rerunning our analyses with the sum scores of the CESD (depression) and STAI (anxiety) questionnaires added to our models.

## RESULTS

### Sample Description

As shown in Table 1, mothers had an average age of 25.2 years (SD = 4.55; range 18.2-37.1 years). Mean age of fetuses was 32.6 weeks (SD = 4.1; range 21-40 weeks) GA at the time of fetal fMRI measurement. Women’s cumulative CTQ scores ranged from 25 to 113, and 43 women (48.3%) reported exposure to at least 1 type of moderate-to-severe childhood maltreatment. We mean imputed 12 cases (13.5%) because of missing values on 1 item. These cases did not significantly differ on any predictors compared to cases without missing data (CTQ scores: *t* = 0.561, *p* = .577; NASF scores: *t* = −0.188, *p* = .852). Two-sample *t* tests and χ^2^ tests showed that maternal maltreatment status was not related to fetal age at scan, GA at birth, maternal NASF scores, separate scores on the NASF questionnaires, maternal depression at 3 years postpartum, motion values, frame count, income, maternal education, or health status (Table 1). However, mothers who reported CM vs those who did not were on average younger (mean = 23.7 vs 26.5 years; *t* = 3.086, *p* = .003), and Black American women were more likely than White women to be in the CM+ group (χ = 7.673, *p* = 0.022). In addition, mothers in the CM+ group reported higher anxiety levels at 3 years postpartum as compared to the CM– group (*t* = −2.354, *p* = .021).

### Fetal Amygdala Neurocircuitry

**FIGURE 1:**
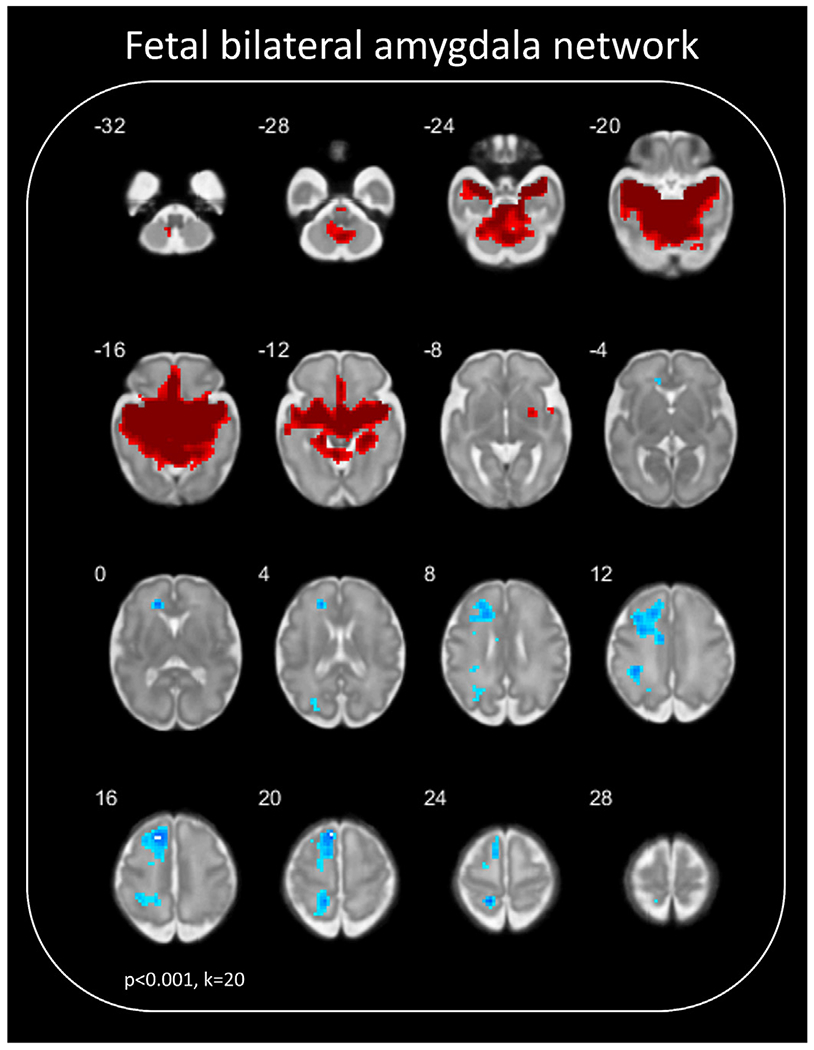
Analyses of Whole Brain Amygdala Connectivity ***Note:*** *A one-sample t test highlights whole brain amygdala (bilateral) connectivity for the full sample (N = 89), controlling for gestational age at scan. Red coloring refers to positive connectivity, whereas blue coloring refers to inverse connectivity. Here, we see strong significantly positive connectivity between the amygdala and cerebellum, the insula, and the medial and lateral temporal lobes. We also note significantly inverse connectivity to the left superior frontal gyrus, left visual association areas, left somatomotor cortex, and left prefrontal cortex (PFC). Results are displayed on a 32-week gestational age cortical surface*^37^ *at* p < 0.001, k > 20.

Fetal amygdala connectivity (*p* < .001 and *k* > 20) is displayed in Figure 1. Across the full sample, the fetal amygdala network comprised strong positive connectivity to ventromedial prefrontal cortext (vmPFC), cerebellum, insula, and the medial and lateral temporal lobes. In contrast, regions of the left superior frontal gyrus, left visual association areas, left somatomotor cortex, and left prefrontal contex (PFC) showed inverse patterns of connectivity. Positive connectivity was predominately bilateral, whereas inverse connectivity was predominately localized in the left hemisphere. When examining fetal amygdala connectivity with a more liberal threshold (*p* > .05 and *k* > 20), a similar pattern emerged (see Figure S3, available online).

### Maternal CM and Fetal Amygdala Connectivity

**FIGURE 2:**
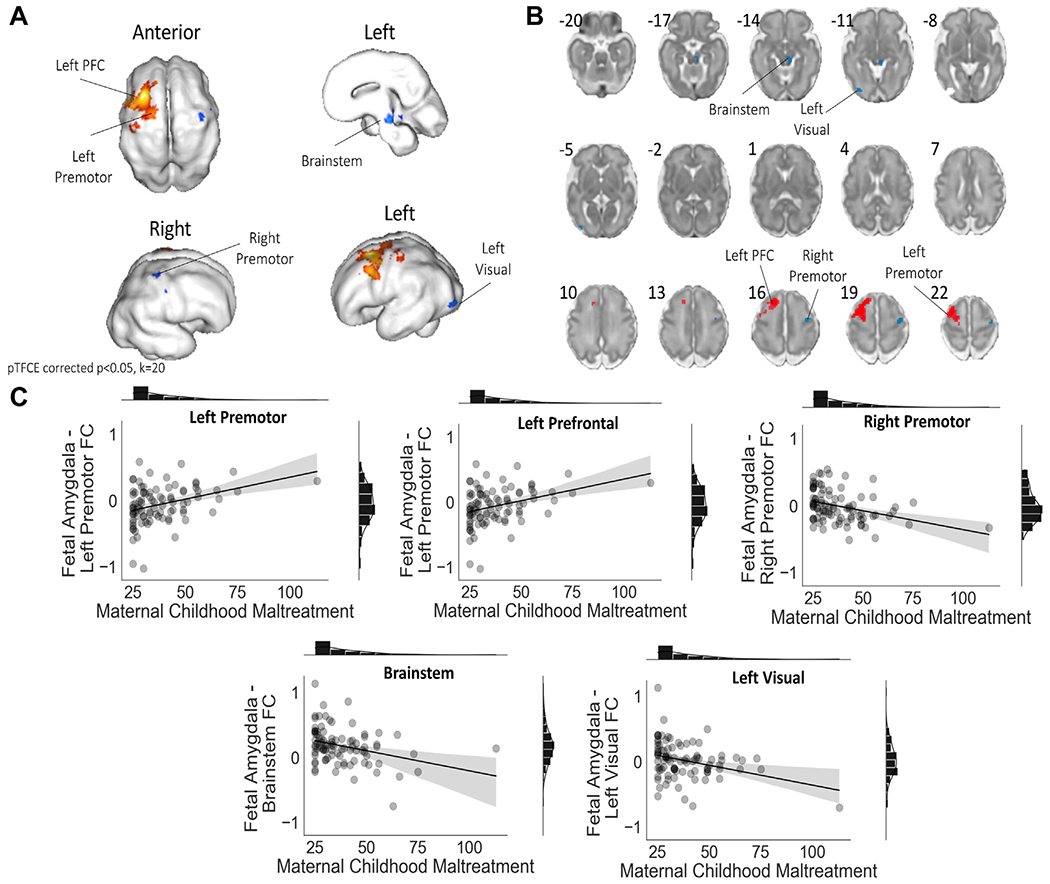
Maternal Childhood Trauma Is Related to Differences in Fetal Whole Brain Amygdala Functional Connectivity ***Note:*** *T-score contrast maps from regression analysis of log transformed maternal Childhood Trauma Questionnaire (CTQ) summary scores with whole brain amygdala connectivity are displayed on a 32-week gestational age cortical surface*^37^ *in 3 dimensions (A) and on cortical slices (B). These images are pTFCE corrected* p < *0.05 and* k >*20. The lower panel (C) shows scatterplots including a linear regression line and distribution of each outcome, per brain area. We observed relatively more amygdala connectivity to left prefrontal cortex (PFC) and left premotor, right premotor and relatively less amygdala connectivity to brainstem, and left visual cortex. Results remained significant after deletion of 2 outliers, except for the amygdala to left visual result (became non-signficant)*.

CM severity was associated with altered connectivity in the amygdala network in several brain regions (Figure 2). Specifically, in fetuses of mothers with greater CM severity, we found relatively higher amygdala connectivity to the left prefrontal cortex (PFC) and left premotor area, as well as relatively less amygdala to brainstem, left visual cortex, and right premotor area connectivity. These effects remained significant when controlling for maternal NASF, fetal sex, motion variables, frame count, maternal education, maternal income, and maternal age at scan as covariates. Table 2 (Steps 1 and 2) reports the results of the regression, and Figure 2 provides an overview of significant brain areas and scatterplots. Regression models are presented only with statistics for CM severity, fetal sex, and NASF; other covariates are not displayed because of space concerns. For a full model, including statistics for all covariates, see Tables S1 to S5, available online.

### Sex Effects

Comparing male and female fetal amygdala connectivity in our 5 significant clusters, there were no significant sex differences (all *p* values >.05). The differential effect of CM on fetal brain connectivity by fetal sex [log(CTQ) by sex interaction] was only significant for the amygdala to the brainstem correlation (*t* = −2.109, *p* = .039). Simple slope analyses showed that the association between maternal CM on fetal amygdala to brainstem connectivity was marginally significant in female individuals (*t* = −1.803, *p* = .090) and was non-significant in male individuals (*t* = −0.908, *p* = .370), after controlling for confounders. Table 2 (Step 3)<<? provides the results of the regression model including the sex interaction.

### Sensitivity Analyses

We first excluded 2 outliers (1 extreme CTQ score, 1 extreme RSFC score) and re-ran our models. This produced similar findings, with 2 exceptions. First, the association between maternal CM and amygdala to left visual cortex connectivity became non-signficant. Second, the sex interaction for the amygdala to the brainstem became non-significant. We then re-ran our models with dichotomized CTQ scores, which resulted in similar but weaker effects. Third, we found that maternal CM scores were not correlated with average translational (*r* = 0.112, *p* = .295) or rotational (*r* = 0.118, *p* = .269) motion or with frame count (*r* = 0.152 , *p* = 0.155). Next, none of our RSFC values associated with motion or frame count (see Tables S1 to S5, available online), suggesting that systematic differences in fMRI quality measures, which have the potential to confound stress–brain associations, are unlikely to explain the reported results. Finally, we checked whether controlling for maternal postnatal distress (anxiety and depression) affected our results. Fetal amygdala functional connectivity in all brain regions was still significantly associated with maternal CM (all *p* < .05), except for the brainstem region (*t* = −1.926, *p* = .059).

## DISCUSSION

In this study, pregnant women’s experiences of childhood maltreatment were reflected in offspring brain amygdala network connectivity in utero. Based on a sample of low-SES, predominantly Black American women, mothers’ greater exposure to childhood maltreatment was associated with variation in fetal functional connectivity between the amygdala and key brain regions involved in emotion regulation networks and sensorimotor and perceptual processing. Sex differences were examined, and, contrary to some reports on prenatal exposures and early brain development,^23,44^ they were absent. However, interactions may be underpowered because of our modest sample size. In addition, we report on general amygdala functional connectivity in utero, showing that the amygdala and prefrontal areas are already functionally connected in late gestation. Our findings add to prior work suggesting that women’s adverse experiences from their own childhoods can alter their offspring’s neurodevelopmental trajectories with implications for psychiatric disease risk,^45^ yet, similar to a few other reports,^13–15^ they isolate the timing of influence to biological processes active in the intrauterine or even pre-conception period.

### Altered Fetal Amygdala FC Following Maternal Childhood Maltreatment

Overall, fetuses of mothers exposed to maternal childhood maltreatment manifest relatively more functional connectivity of the amygdala to the left prefrontal and premotor areas, and relatively less functional connectivity of the amygdala to the brainstem and the right premotor cortex. The extent of frontoamygdala effects extended over a large part, mostly medial, of the prefrontal and premotor area. This finding is consistent with Hendrix *et al.*,^14^ who also reported stronger connectivity of the left amygdala and mPFC in newborns of mothers exposed to childhood maltreatment. Our study extends prior research by establishing that maternal childhood maltreatment may influence offspring’s amygdala–PFC network development before birth. Taken together, our report and those of others show that the amygdala–PFC network may be altered starting in the womb and that this alteration persists into infancy. Although the long-term functional significance of these neural alterations remains unclear, more positive coupling between the amygdala and dACC is associated with heightened threat sensitivity and anxiety disorders in children^46^ and adults.^47^ Future longitudinal work is required to determine whether altered frontoamygdala circuitry in utero is associated with similar behavioral outcomes.

Interestingly, we observed lateralization of effects, with stronger effects of maternal childhood maltreatment experiences in the left hemisphere. Two other studies on the effect of maternal childhood maltreatment on newborn brain development also reported stronger effects for the left hemisphere compared to the right.^13,14^ In addition, we showed left lateralization of fetal amygdala functional connectivity. Whether left lateralization of amygdala connectivity and lateralization of the effect of maternal CM are related is unclear. It could be that, because of its relative faster growth during fetal brain development, the left hemisphere is more vulnerable to prenatal exposures, such as changes in gestational biology related to maternal maltreatment history.^48^ Several fetal MRI and sonography studies have shown that left laterialization starts in utero, by identifying a larger left hemisphere and larger gray matter volumes on the left side.^48^ Very recently, a fetal resting-state fMRI study demonstrated lateralization in the fetal connectome, with left lateralization in the prefrontal cortex.^49^ Together with our results, these findings indicate that functional connectivity patterns may already lateralize during prenatal brain development.

### Potential Effect of Maternal Prenatal Distress

Our study examined whether maternal psychological distress during pregnancy, including depressive symptoms, anxiety, worrying, and lower quality of life, affected the association between maternal childhood trauma and fetal amygdala connectivity as a confounder. Maternal psychological stress during pregnancy did not significantly contribute to the model, and all effects of maternal CM remained significant when controlling for maternal psychological distress during pregnancy. This seems to indicate dissociable impacts of childhood maltreatment that yield effects independent of prenatal distress. Our finding is in line with previous reports on the effects of maternal CM on offspring brain volume^13,15^ and on newborn functional connectivity,^14^ in which the effects of maternal CM also remained significant after controlling for maternal prenatal (psychological) distress. Notably, unlike Moog *et al.*^13^ and Hendrix *et al.*,^14^ we did not find higher maternal prental distress levels in women exposed to CM as compared to those without CM. This could potentially be due to the high levels of prenatal distress in all of the mothers in our high-risk sample. The women in our sample are predominantly single mothers, with a low income, with approximately 60% having an annual income below $20,000.

### Fetal Sex as Moderator

We did not find any interactions with fetal sex for the association between maternal CM and fetal amygdala function connectivity. However, with our modest sample size, interactions may be underpowered, and/or outlier influences can exert unintended effects. We emphasize that our results regarding fetal sex are preliminary and mainly intended to aid in hypothesis generation. Future investigation of sex differences in larger prenatal neuroimaging studies is certainly warranted.

### Mechanisms of Intergenerational Transmission

Several potential pathways by which mothers’ childhood experiences may affect the next generation prior to birth and independent of current distress have been suggested.^9,10^ First, CM-related epigenetic alterations in the maternal germline that survive re-establishment of post-conception epigenetic alterations are a potential mechanism of transmission. CM-related paternal epigenetic alterations are less well explored, but could also play an important role, given that women with high CM have a higher chance of pairing with CM-exposed men, based on the principles of “assortative pairing.”^50^ Second, CM may lead to changes in the maternal oocyte cytoplasm (such as to mitochondria) that in turn influence her developing embryo. Third, from a life course perspective, researchers have suggested that CM-associated changes in maternal gestational biology, such as atypical endocrine (often hypothalamic–pituitary–adrenal [HPA] axis) or immune-inflammatory regulation may shape fetal development.^51^ These changes are intrauterine perturbations that the fetal–placental unit “senses” and to which the fetus adapts, leading to alterations in anatomy and/or physiology such as decreased neurogenesis, variation in neuronal migration, and formation of synapses.^52^ In the emerging field of intergenerational transmission, 1 report showed that maternal CM was associated with an almost 25% increase in placenta-derived maternal peripheral blood corticotrophin-releasing hormone levels with implications for birth age, weight, and temperament,^53^ and our group found that maternal CM was associated with a reduction in fetal heart rate variability, an index of less adaptive autonomic nervous system development and relevant for future emotion regulation.^54^ Finally, inherited psychiatric risk should not be excluded as a potential pathway, as parents with psychiatric illnesses are more likely to maltreat their children.^55^ Alterations in fetal functional connectivity may be (also) related to this inherited psychiatric risk. We do want to emphasize, however, that parents at psychiatric risk should not be stigmatized—the fact that a parent has a psychiatric illness does not mean that they will maltreat their child(ren).

### Amygdala Functional Connectivity In Utero

Across all fetuses, on average 32 weeks old, and controlling for gestational age at scan, there were robust findings of functional connectivity between the bilateral measurements of the amygdala and key brain regions. Specifically, we identified significantly positive connectivity between the amygdala and cerebellum, insula, and the medial and lateral temporal lobes. We also found significantly inverse connectivity to the left temporal junction, left superior frontal gyrus, left visual association areas, left somatomotor cortex, and left PFC. These connectivity patterns are consistent with other imaging studies showing the emerging connectome in neonates and preterm infants^56^ and at 6 months postnatal.^51^ Data presented here show that, although immature and potentially weakly structurally connected, the amygdala and prefrontal areas are already functionally connected in late gestation. Notably, all inverse connectivity reported in our study was left lateralized, indicating a potential left lateralization of inverse amygdala functional connectivity in fetuses. Continued research is necessary to map the developmental trajectory of amygdala connectivity in early life, starting in utero.

Potential limitations of the study warrant mention. First, the present study did not use subject- specific anatomical regions of interest, as availability of reconstructed volumetric data for these cases is scarce. Consequenlty, potential individual and age-related variation in amygdala structure is not captured in our approach. We expect that variation present would have minimal influence on our approach, because the amygdala size paired with our functional image resolution (3.4 mm isotrophic) is not sensitive to individual anatomical differences. Second, the main predictor in our study, maternal childhood trauma, was collected using a retrospective questionnaire at child age 3 years. Retrospective accounts may unduly reflect both recent events (eg, early parenting and/or birth experiences), and unresolved emotional trauma stemming from childhood years. As such, retrospective measures of child maltreatment may carry additional information about resilience and/or later life experiences. It is a strength of our study that the rates of childhood maltreatment in our sample are very similar to those in previous studies investigating the effect of maternal CM on the offspring’s brain in similar samples (45% of mothers).^14^ Third, negative experiences in adolescence or adulthood, such as low social support, intimate partner violence, and stressful life events, may be more frequent in those mothers exposed to childhood maltreatment.^57^ These factors were not taken into account in the current study, due to lack of power of the statistical analyses. Nevertheless, no differences were found between mothers with and without CM for education and family income, suggesting similar experiences in later life in both groups. Fifth, some concerns may be raised regarding the scales that the NASF is based on. Some items of the depression scale (CES-D), for instance, may overlap with pregnancy symptoms, such as feeling tired and having trouble sleeping. Moreover, the trait scale of the State-Trait Anxiety Inventory and the Penn State Worry Questionnaire may measure maternal traits instead of distress during pregnancy. Future research could utilize scales that are more commonly used in the perinatal period, such as the Edinson Postpartum Depression Scale (EPDS).^58^

Another consideration regarding the present study is that our research concentrated on women from under-represented racial and ethnic groups and women from low-socioeconomic households, raising questions about the generalizability of our results to other samples. It is important that groups less frequently engaged in research be intentionally recruited into studies to close extant gaps,^59^ and there is scientific value in reducing SES variability when exploring topics of CM. In a final note, this study was conducted to test the falsifiable hypothesis that the fetal amygdala network would show altered connectivity in targeted regions, which raises the possibility that additional significant differences in fetal connectivity may be missed here. For instance, the cerebellum seems to be an important area for fetal functional connectivity according to two recent studies from our own group.^26,35^ In the future, there will be opportunity to use alternative approaches and to address additional questions in larger samples, as data from this project and others continue to be quality assured, processed, and released on an ongoing basis.

Taken together, data presented here demonstrate that pregnant women’s experiences of childhood maltreatment may be reflected their offspring’s brain development in utero. Mothers’ exposure to childhood maltreatment was associated with variation in fetal functional connectivity between the amygdala and left prefrontal, left and right premotor areas, and the brainstem. In line with previous work, the largest effects of maternal CM were found in the left hemisphere, suggesting lateralization of CM exposure in the offspring’s brain. No clear sex differences were found in our study, but our modest sample size may not have been sufficient to detect (small) sex effects. Our findings add to prior work suggesting that women’s adverse childhood experience can alter their offspring’s neurodevelopmental trajectories before birth with implications for psychiatric disease, and extend the DOHaD model to include maternal childhood experiences. In particular, this study isolates the timing of influence to biological processes active in the intrauterine or pre-conception period. Our data support current changes in health policy moving toward collaborative care models with a life course perspective in which assessment of childhood maltreatment and mental health screening are routinely integrated into prenatal care—for women, and for their future children.

## Supplementary Material

supplemental

## Figures and Tables

**TABLE 1:** Demographic Characteristics Overall and by Childhood Maltreatment (N = 89)

	Total sample (N = 89)			CM– Group (No childhood maltreatment) (n = 46)			CM + Group (≥1 Type of childhood maltreatment) (n = 43)			Diff between groups
Variables	N	Mean (SD) or %		n	Mean (SD) or %		n	Mean (SD) or %		*p* ^a^
Demographics										
Maternal race/ethnicity										.022
White	8	9.0		8	17.4		0	0		
Black American	74	83.1		36	78.3		38	88.4		
Biracial	4	4.5		2	4.3		2	4.7		
Nondisclosed	3	3.4		0	0		3	7.0		
Maternal age, y	89	25.2	(4.5)	46	26.5	(5.1)	43	23.7	(3.3)	**.003**
Maternal education										.710
No GED/no high school diploma	13	14.6		5	10.9		8	18.6		
GED/high school diploma	32	36.0		19	41.3		13	30.2		
Some college	34	38.2		17	37.0		17	39.5		
2-y college degree	2	2.2		1	2.2		1	2.3		
4-y college degree	3	3.4		2	4.3		1	2.3		
Master’s degree	1	1.1		1	2.2		0	0		
Doctoral degree	1	1.1		1	2.2		0	0		
Gross annual income ($)										.113
<10,000	32	36.0		15	32.6		17	39,5		
10,000-20,000	19	21.3		14	30.4		5	11,6		
20,000-30,000	14	15.7		7	15.2		7	16,3		
30,000-40,000	5	5.6		1	2.2		4	9,3		
50,000-60,000	3	3.4		2	4.3		1	2,3		
60,000-80,000	3	3.4		3	6.5		0	0		
100,000-120,000	2	2.2		2	4.3		0	0		
220,000-250,000	1	1.1		0	0.0		1	2,3		
**Maternal trauma and prenatal distress**										
CTQ Total	89	37.6	(14.5)	46	28.9	(4.4)	43	46.8	(15.8)	<.001
PSS-10 (Perceived stress)	83	15.34	(6.6)	41	15.4	(6.1)	42	15.24	(7.2)	.891
SWLS (Life satisfaction)	84	24.95	(6.4)	42	25.14	(6.6)	42	24.76	(6.2)	.786
STAI (Anxiety)	84	34.98	(8.3)	42	34.6	(8.0)	42	35.33	(8.6)	.695
PSWQ (Worrying)	85	42.33	(12.5)	42	42.19	(11.7)	43	42.47	(13.3)	.920
CES-D (Depression)	86	12.8	(7.5)	45	11.6	(6.9)	41	14.16	(7.9)	.115
NASF (Stress factor)	89	−0.16	(0.9)	46	−0.20	(0.8)	43	−0.13	(0.9)	.731
**Maternal postnatal distress (3 y)**										
STAI (Anxiety)	86	33.63	(8.26)	45	31.67	(7.25)	41	35.79	(8.84)	**.021**
CES-D (Depression)	84	3.74	(5.59)	43	3.24	(4.92)	41	4.27	(6.23)	.400
**Prenatal alcohol and smoking**										
Have 5 or more alcoholic drinks/day										.966
No	83	93.3		42	91.3		41	95.3%		
Yes	3	3.4		2	4.3		1	2.3%		
Missing	3	3.4		2	4.3		1	2.3%		
Smoke cigarettes daily										.343
No	79	88.8		40	87.0		39	90.7		
Yes	6	6.7		3	6.5		3	7,0		
Missing	4	4.5		3	6.5		1	2.3		
Have contact with cigarette smoke										.496
No	41	46.1		18	39.1		23	53.5		
Yes	44	49.4		25	54.4		19	44.2		
Missing	4	4.5		3	6.5		1	2.3		
**Infant outcomes**										
Gestational age at birth, wk	89	39.1	(1.4)	46	39.0	(1.6)	43	39.2	(1.1)	.422
Infant weight at birth, gr	89	3259	(508)	46	3277	(553)	43	3241	(462)	.743
Gestational age at scan, wk	89	32.6	(4.1)	46	33.2	(4.0)	43	31.9	(4.2)	.142
Infant sex										.283
Male	55	61.8		31	67.4		24	55.8		
Female	34	38.2		15	32.6		19	44.2		

**Note**: Boldface p values are significant. CES-D = Center for Epidemiological Studies Depression Scale; CM = childhood maltreatment; CTQ = Childhood Trauma Questionnaire; GED = graduation equivalency degree; NASF = Negative Affectivity and Stress Factor; PSS-10 = 10-item Perceived Stress Scale; PSWQ = ; STAI = State Trait Anxiety Inventory; SWLS = Satisfaction with Life Scale.

a Baseline differences are assessed using t tests for continuous measures and **χ**^2^ test for categorical measures.

**TABLE 2:** Regression Models of Maternal Childhood Maltreatment (CM) and Fetal Amygdala Brain Connectivity

		B	SE	95% CI b	*p*	R^2^	ΔR^2^	df
Right premotor	Step 1: Select covariates					0.13	0.13	0.71
	Fetal sex	−0.02	0.06	−0.95, 0.13	.72			
	NASF	0.06	0.04	−0.01, 0.13	.09			
	Step 2: Main effect					0.33	0.20	16.81
	log(CTQ)	−0.32	0.08	−0.48, −0.17	<**.001**			
	Step 3: Interaction					0.35	0.02	1.81
	log(CTQ)×Fetal sex	−0.22	0.17	−0.57, 0.11	.18			
Left visual region	Step 1: Select covariates					0.13	0.13	0.73
	Fetal sex	−0.06	0.08	−0.21, 0.09	.43			
	NASF	0.03	0.05	−0.06, 0.13	.47			
	Step 2: Main effect					.21	0.08	5.58
	log(CTQ)	−0.27	0.11	−0.50, −0.04	**0.02**			
	Step 3: Interaction					.22	0.01	0.69
	log(CTQ)×Fetal sex	0.20	0.24	−0.29, 0.69	0.41			
Brainstem	Step 1: Select covariates					.18	0.18	1.07
	Fetal sex	−0.07	0.07	−0.22, 0.08	0.36			
	NASF	−0.02	0.05	−0.11, 0.07	0.64			
	Step 2: Main effect					.24	0.06	4.48
	log(CTQ)	−0.23	0.11	−0.45, −0.01	**0.04**			
	Step 3: Interaction					.32	0.08	6.53
	log(CTQ)×Fetal sex	−0.59	0.23	−1.04, −0.13	0.01			
Left PFC	Step 1: Select covariates					.12	0.12	0.64
	Fetal sex	−0.03	0.07	−0.17, 0.12	0.68			
	NASF	0.06	0.04	−0.03, 0.15	0.16			
	Step 2: Main effect					.23	0.11	7.86
	log(CTQ)	0.29	0.11	0.08, 0.50	<**0.01**			
	Step 3: Interaction					.24	0.02	1.40
	log(CTQ)×Fetal sex	0.27	0.23	−0.19, 0.72	0.24			
Left premotor	Step 1: Select covariates					.13	013	0.71
	Fetal sex	−0.01	0.07	−0.15, 0.13	0.86			
	NASF	0.01	0.04	−0.07, 0.10	0.75			
	Step 2: Main effect					.29	0.16	12.98
	log(CTQ)	**0.35**	0.10	0.16, 0.55	<**0.001**			
	Step 3: Interaction					.29	0.00	0.00
	log(CTQ)×etal sex	0.01	0.21	−0.42, 0.44	0.96			

**Note**: *All models are additionally controlled for maternal age at scan, maternal race, maternal education, family income, maternal smoke exposure during pregnancy, translational and rotational motion, and frame count in step 1 of each model. Select covariates are displayed in this table to enhance readability, but* Tables S1 to S5, *available online, display the association between all covariates with fetal amygdala brain connectivity. All models were run with 5,000 bootstrapped samples; Boldface* p *values are significant. B* = *unstandardized beta; CTQ* = *Childhood Trauma Questionnaire (total sum score); df* = *degrees of freedom; NASF* = *Negative Affectivity and Stress Factor; PFC* = *prefrontal cortex; SE* = *standard error*.
