# Supplementary material for: Intergenerational Transmission of Maternal Childhood Maltreatment Prior to Birth: Effects on Human Fetal Amygdala Functional Connectivity
Source: J Am Acad Child Adolesc Psychiatry. Author manuscript; Available in PMC 2024 Feb 6. (PMC10845129; doi:10.1016/j.jaac.2023.03.020)

**SUPPLEMENTAL MATERIAL**

**Intergenerational Transmission of Maternal Childhood Maltreatment Prior to Birth: Effects on Fetal Brain Functional Connectivity**

van den Heuvel, M.I., Monk, C., Hendrix, C., Hect, J.L., Lee, S., Feng, T., Thomason, M.E. (2023) Intergenerational transmission of maternal childhood maltreatment prior to birth: Effects on human fetal amygdala functional connectivity. Journal of the American Academy of Child and Adolescent Psychiatry. 62(10):1134-1146.

PMID: 37245707

**Supplemental Figures**

**
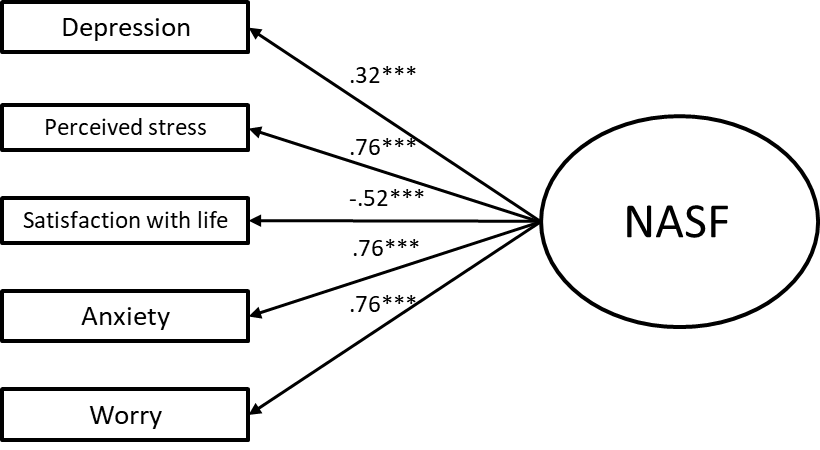
**

**Figure S1. Graphical representation of NASF including factor loadings.** All five scales show high loadings and good fit to a one-factor model (n = 99, CFI = .98, TLI = .97, RMSEA = .06, SRMR = .03; factor loadings, *p* < .001). Note: NASF = Negative Affect and Stress Factor. ∗p < .05; ∗∗p < .01; ∗∗∗p < .001.

**
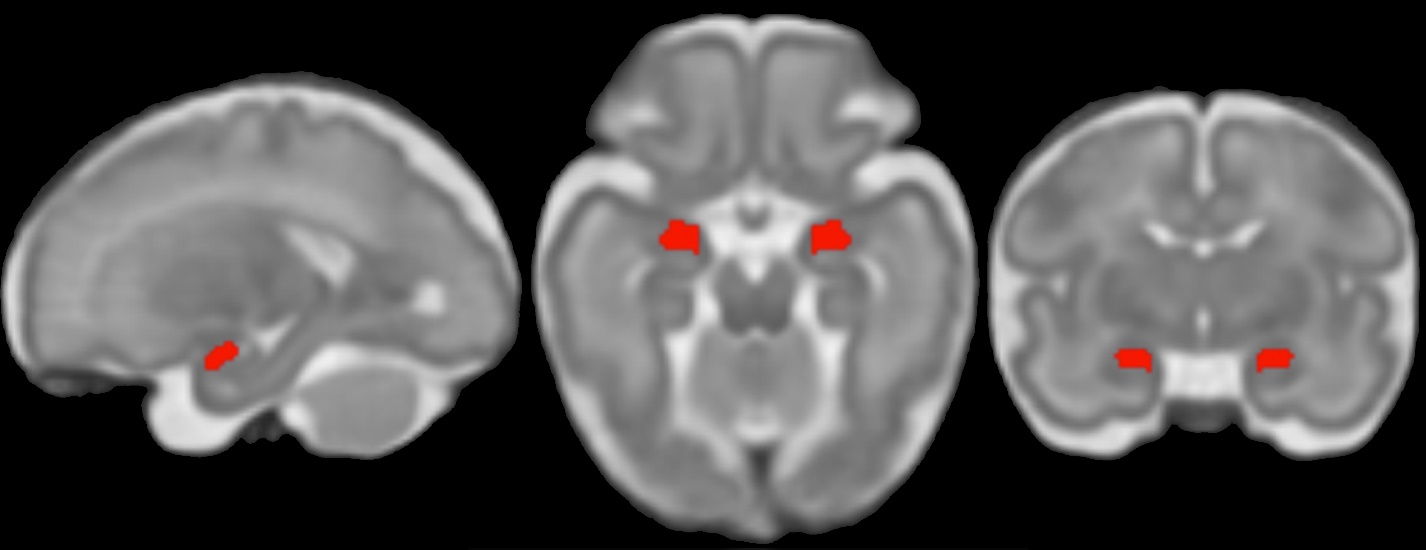
**

**Figure S2. Amygdala ROI seed regions.** Subject level amygdala connectivity maps were generated in CONN toolbox first level analysis from bilateral amygdala ROI seed regions (red). To generate these masks, the amygdala was hand traced on a 32-week gestational age template (61), respecting anatomical boundaries, for the left hemisphere and was then mirrored onto the right hemisphere.

**
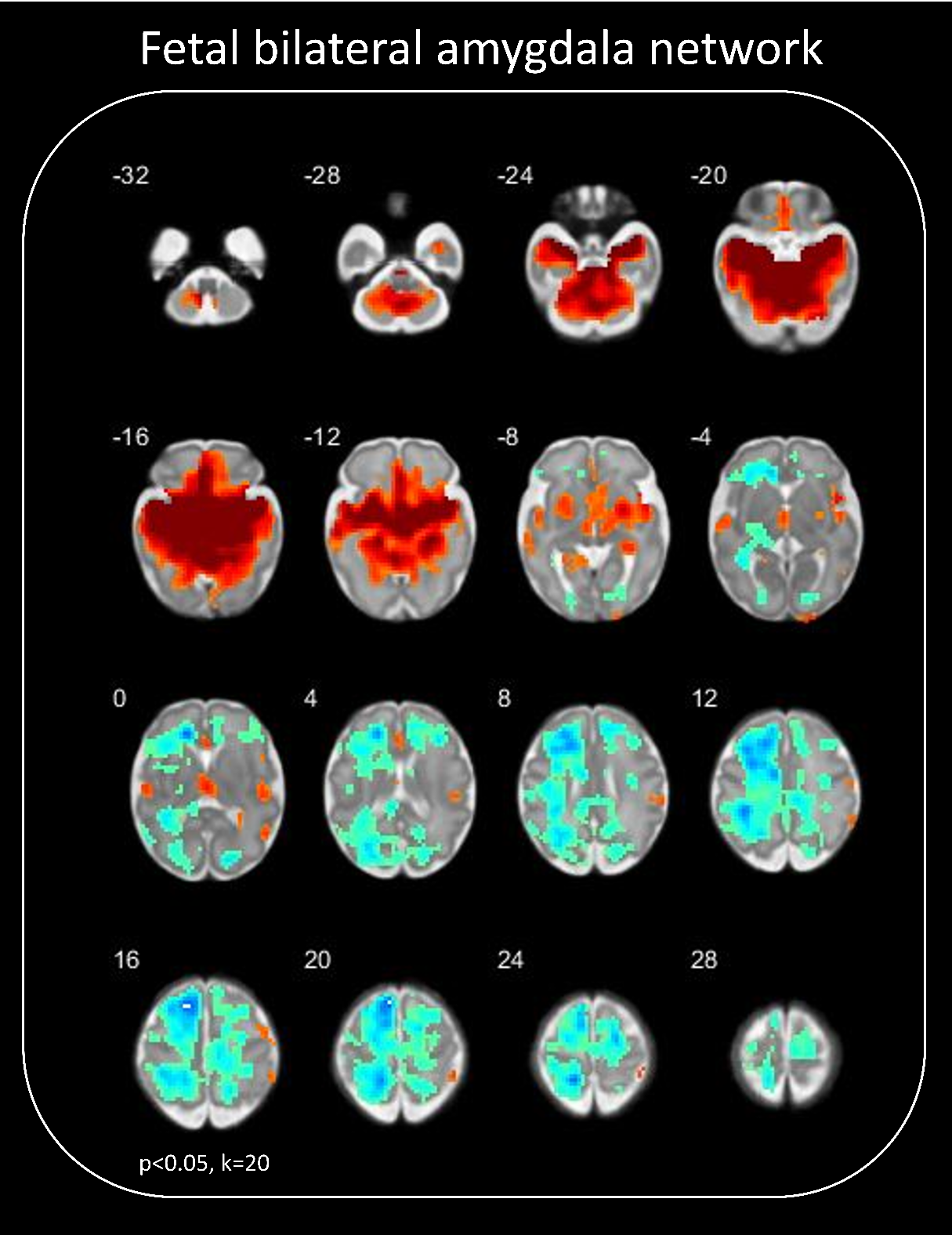
**

**Figure S3. Analyses of whole brain amygdala connectivity.** A one sample *t*-test highlights whole brain amygdala (bilateral) connectivity for the full sample (N=89), controlling for gestational age at scan. Here, we see strong significantly positive connectivity between the amygdala and cerebellum, insula, and the medial and lateral temporal lobes. We also note significantly inverse connectivity to the left superior frontal gyrus, left visual association areas, left somatomotor cortex, and left prefrontal cortex (PFC). Results are displayed on a 32-week gestational age cortical surface^35^ at *p* < 0.05, k > 20.

**Table S1 Regression analyses for Right premotor region**

**
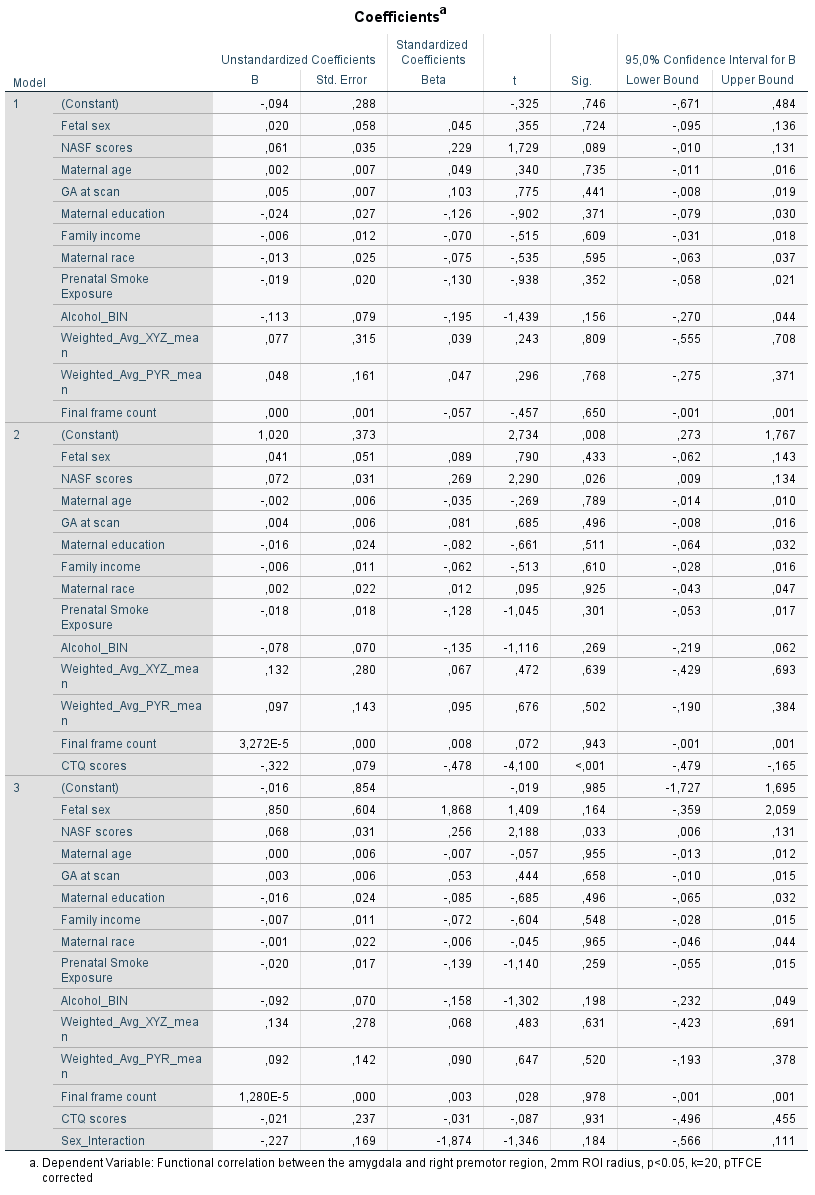
**

**Table S2. Regression analyses for Left visual region**


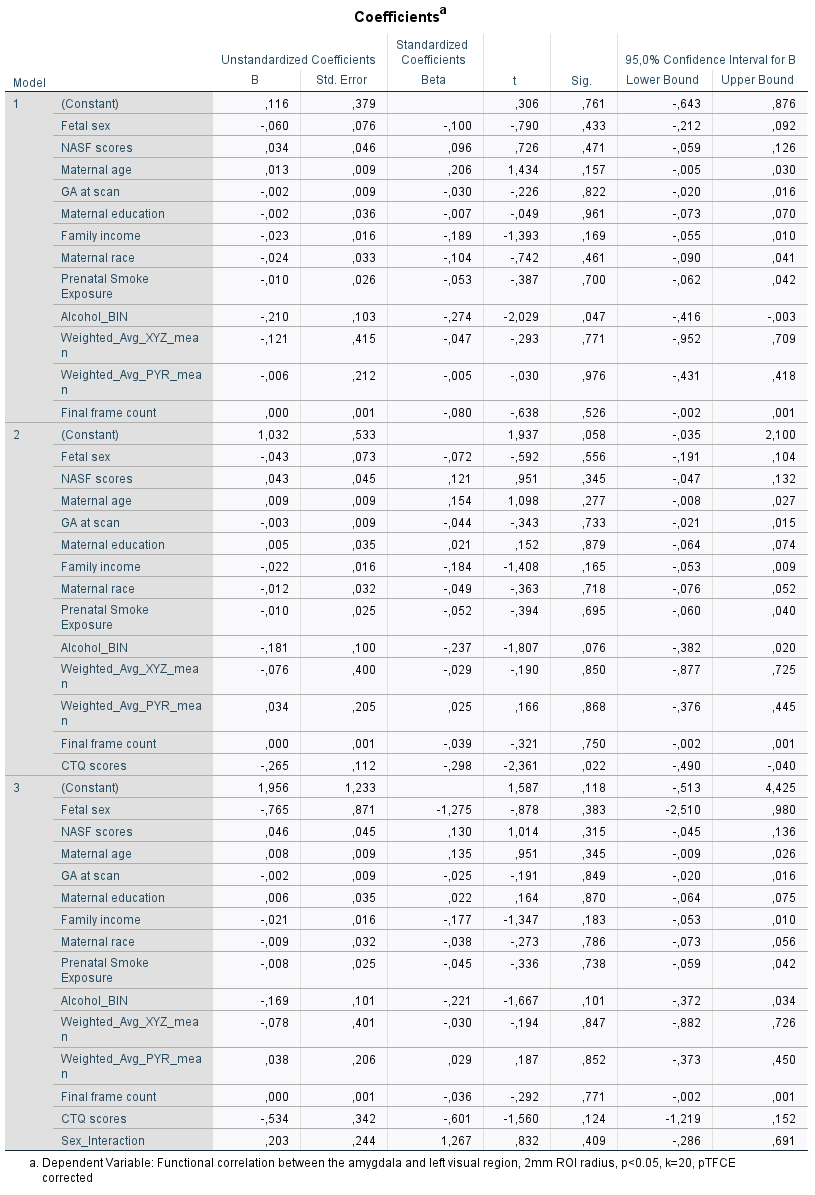


**Table S3. Regression analyses for Brainstem**

**
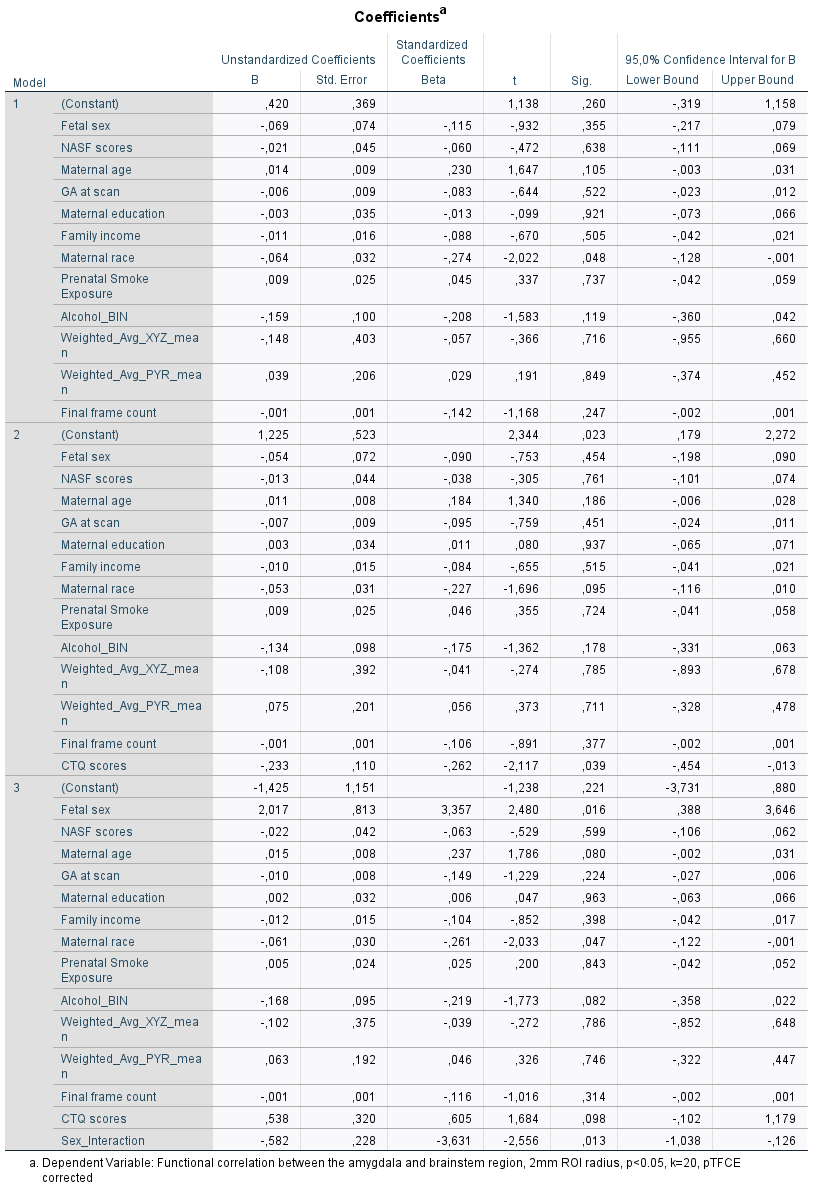
**

**Table S4. Regression analyses for Left PFC**

**
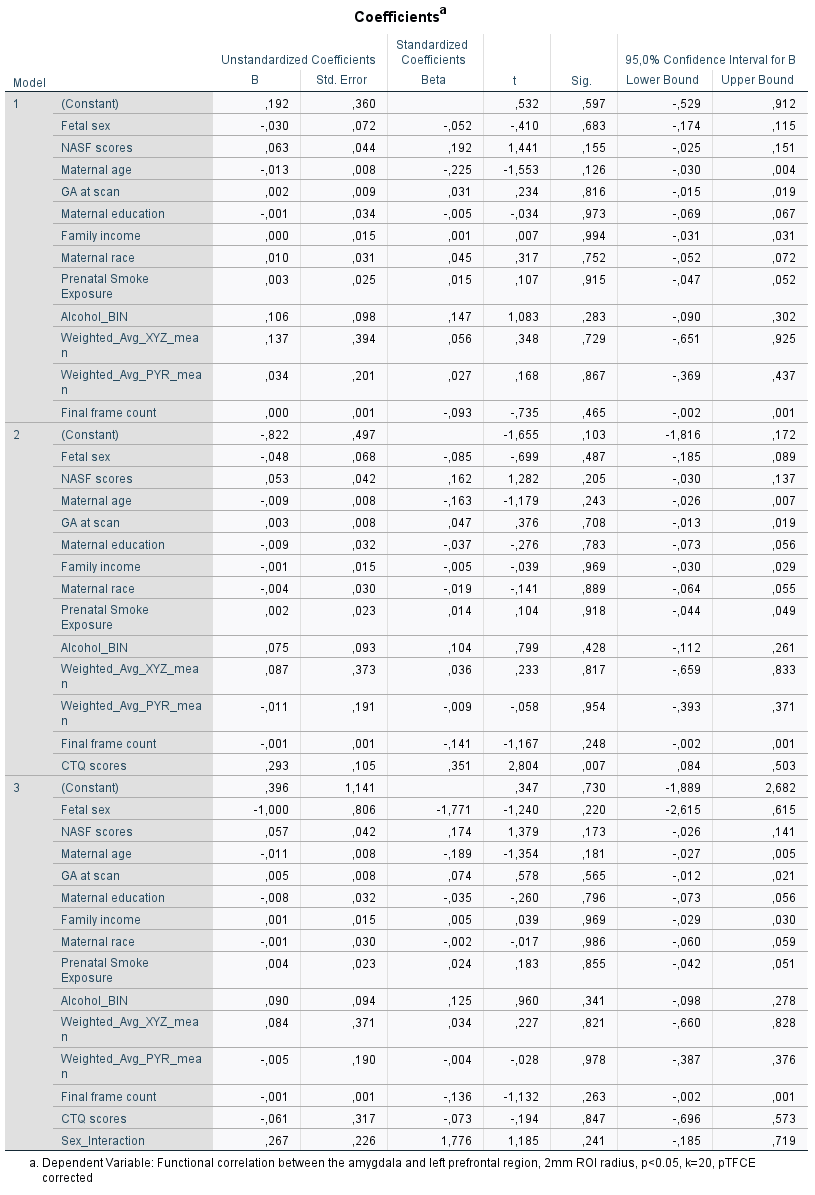
**

**Table S5. Regression analyses for Left premotor**
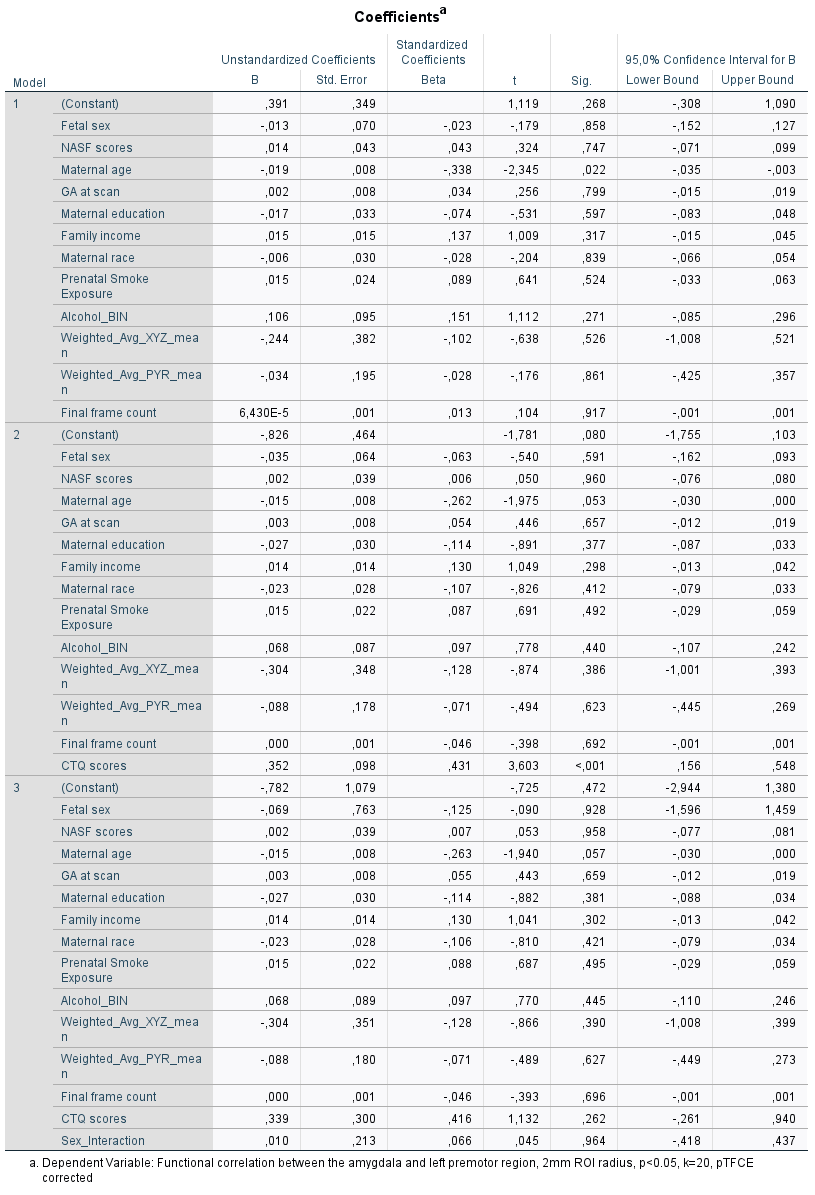

Supplement: supplemental [file NIHMS1957029-supplement-supplemental.docx]
